# Impact of HIV on Neuronal Health in the Brains of People with HIV

**DOI:** 10.3390/v18090999

**Published:** 2026-09-11

**Authors:** Thomas A. Angelovich, Jessica J. Kivinen, Sarah J. Byrnes, Paul R. Gorry, Melissa J. Churchill

**Affiliations:** 1 ATRACT Centre, School of Health and Biomedical Sciences, RMIT University, Bundoora, VIC 3083, Australia; thomas.angelovich@rmit.edu.au (T.A.A.); sarah.byrnes@rmit.edu.au (S.J.B.); 2 Life Sciences Discipline, Burnet Institute, Melbourne, VIC 3004, Australia; 3 Department of Infectious Diseases, The Peter Doherty Institute for Infection and Immunity, The University of Melbourne, Melbourne, VIC 3000, Australia; 4 Department of Microbiology and Immunology, The Peter Doherty Institute for Infection and Immunity, The University of Melbourne, Melbourne, VIC 3000, Australia; prgorry@unimelb.edu.au

**Keywords:** HIV, neuroinflammation, reservoirs, neurons, dysfunction

## Abstract

Despite effective viral suppression with antiretroviral therapy (ART), people with HIV (PWH) remain at increased risk of neurocognitive impairment and persistent neuroinflammation. Although neurons are not infected by HIV, they remain susceptible to the indirect effects of chronic infection, including exposure to viral proteins, inflammatory mediators, and glial dysfunction. The resulting neuronal injury is thought to contribute to the development and persistence of HIV-associated neurocognitive disorders in the ART era. This review examines the impact of chronic HIV infection on neuronal function, highlighting mechanisms of neuronal dysfunction driven by both viral-dependent and viral-independent pathways. We discuss the role of central nervous system viral reservoirs, neuroimmune interactions, and the neurotoxic effects of HIV proteins including Tat, gp120, and Nef. We further explore how chronic HIV infection influences synaptic integrity, neuronal metabolism, and age-related neurodegeneration. Finally, we highlight emerging biomarkers and therapeutic strategies aimed at preserving neuronal function. By integrating current evidence on both viral-dependent and viral-independent mechanisms of neuronal dysfunction, this review identifies critical knowledge gaps and future research priorities aimed at preserving neuron function and improving neurological outcomes in PWH.

## 1. Introduction

Despite viral suppression with antiretroviral therapy (ART), people with HIV (PWH) continue to experience elevated risk of neurocognitive disorders, neuropathology and neuroinflammation. Whilst neurons are not infected with HIV, they are impacted by infection of neighboring cells of the central nervous system (CNS). The impact on neurons is thought to be facilitated through a bystander model in which HIV infects or persists in brain myeloid-lineage cells and trafficking T cell populations, and neuronal dysfunction occurs through exposure to viral proteins, inflammatory mediators, excitotoxic signaling, oxidative stress, and altered trophic support [1]. HIV enters the CNS early following infection [2], and this early viral exposure may establish inflammatory and reservoir dynamics that impact later neuronal outcomes and volumetric changes, even following ART-suppression of viraemia [3,4]. This framework explains how significant neuronal injury can occur even when neurons are not infected, and why the location of the virus fails to always match the pattern of cognitive dysfunction.

In ART-suppressed PWH, neuronal dysfunction persists despite suppression of systemic viral replication and manifests as chronic synaptic, dendritic, axonal, metabolic, and network abnormalities [5,6,7]. It follows that neuronal function in ART-suppressed PWH is likely determined by both legacy injury from earlier uncontrolled infection and potentially due to ongoing low-grade transcription in the CNS. How HIV impacts neuronal integrity in the context of extended ART-suppression and whether those effects are reversible is currently being explored.

In this review, current evidence describing the impact of chronic HIV infection on neuronal health and function will be discussed. Moreover, the implications of chronic neuronal dysfunction for an HIV cure will be addressed.

## 2. Clinical Evidence of Neuronal Dysfunction in PWH

Chronic HIV infection is traditionally associated with neuropathogenesis and cognitive impairment with the clinical correlates of impaired neuronal health in PWH most discussed under the framework of HIV-associated neurocognitive disorders (HAND) and/or HIV-associated brain injury (HABI) [8,9]. Based on these criteria, the reported prevalence of neurocognitive impairment ranges from 10 to 30% depending on the criteria used [8,10,11,12,13]. Specifically, the Frascati criteria define HAND into three distinct categories; asymptomatic neurocognitive impairment (ANI), mild neurocognitive disorder and HIV-associated dementia [8]. In the ART-treated era, PWH with severe dementia is uncommon; however milder and fluctuating impairments affecting attention, executive function, memory retrieval, processing speed, and daily functioning are still common [10]. The inclusion of ANI in particular is controversial as it may over-estimate the true incidence of cognitive impairment and/or the contribution HIV plays which may instead reflect other factors including aging, drug use, co-infections and comorbidities [14]. These changes likely reflect ongoing vulnerability in fronto-striatal, limbic, and white matter circuits rather than one specific type of brain injury. Neuronal dysfunction affects more than just cognition. Mood changes, fatigue, decreased motivation, slowed motor function, and reduced coping ability may also reflect the cumulative effects of HIV-related neuronal and network stress, particularly in older adults and in people with other vascular or psychiatric conditions [15].

It is important to recognize that the literature is divided on the long-term impacts of HIV on the brain with some studies continuing to show chronic impacts despite ART, whilst other studies report a declining prevalence of neurocognitive disorders in well managed ART-suppressed PWH [16,17,18]. Recent evidence in ART-suppressed PWH who initiated therapy at earlier timepoints post-infection has led to a division in the field regarding the specific contribution related to ongoing damage and/or legacy impacts.

## 3. Cellular and Molecular Mechanisms of Neuronal Injury

**Figure 1:**
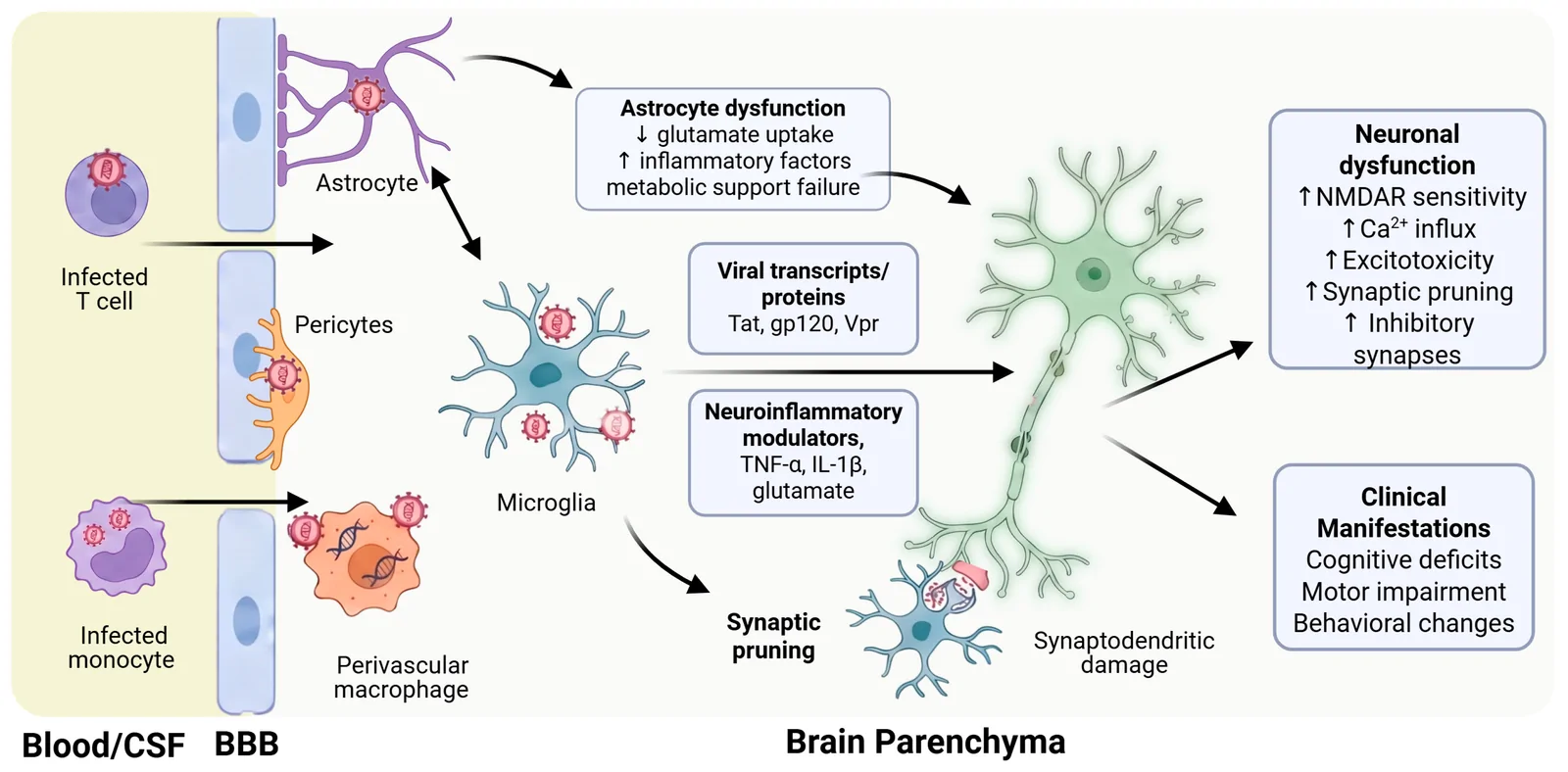
Impact of HIV infection on the brain microenvironment and cognitive function. HIV-infected CD4+ T cells and monocytes traffic across the blood–brain barrier (BBB) via the cerebrospinal fluid (CSF), contributing to infection of resident CNS cells, particularly microglia and, to a lesser extent, astrocytes and pericytes. Although neurons are not productively infected by HIV, viral proteins and inflammatory mediators released by infected and activated cells can induce neuronal dysfunction through mechanisms including oxidative stress, excitotoxicity, N-methyl-D-aspartate receptor (NMDAR) sensitivity, altered synapse balance, elevated synaptic pruning and chronic neuroinflammation. These processes contribute to synaptic and neuroaxonal injury, disruption of the brain microenvironment, and ultimately cognitive impairment in ART-suppressed people with HIV (PWH). Created in BioRender. Angelovich, T. (2026) https://BioRender.com/vtue5j9.

Understanding the mechanisms response for the clinical manifestations of chronic HIV is essential for determining whether neuronal dysfunction reflects ongoing injury, legacy effects from acute infection or a combination of both (Figure 1).

### 3.1. Direct Viral Protein Toxicity on Neurons

Several HIV proteins including gp120, trans-activator of transcription (Tat) and nef have been demonstrated to be neurotoxic and are strongly implicated in the pathogenesis of HIV-associated neuronal injury. These proteins disrupt glutamatergic signaling, dysregulate intracellular calcium homeostasis, impair mitochondrial function, promote oxidative damage, and activate transcriptional pathways associated with stress responses and apoptosis [19,20]. Despite effective suppression of plasma viral replication, low-level expression of viral products, including HIV proteins and transcripts, in the CNS are correlated with ongoing neuroinflammation and may be sufficient to sustain neuronal stress responses [21,22].

HIV Tat plays a key role in enhancing HIV transcription and has therefore been investigated for its latency reversing abilities. However, Tat has several documented neurotoxic roles in the CNS. During the HIV replication cycle in infected cells Tat is released which can interact with surrounding cells, traffic throughout the brain via synaptic transport mechanisms, leading to altered synaptic function, calcium signaling, and mitochondrial integrity in neurons [19,21,23,24,25]. Specifically, Tat binds low-density lipoprotein-related protein on neurons leading to endocytosis and dysfunction of endolysosomes. These processes increase pH which can inhibit autophagy leading to neuronal dysfunction and ultimately neuronal cell death [26]. Upregulation of Tat in a Tat-inducible animal model identified amyloid-β accumulation in neurons, a key factor present in Alzheimer’s disease, which was further confirmed to induce endolysosomal dysfunction in vitro by the addition of Tat to neurons [27]. Oligomerization of Tat and interaction with amyloid-β peptide fibrils was associated with weakened membrane integrity and neuronal toxicity [28]. A study in both SIV-infected non-human primates as well as in vitro models demonstrated that Tat induces intracellular effects including translocation of the transcription factor forkhead box O3 associated with cell stress, aging and DNA repair to the nucleus which triggers neuronal apoptosis [29]. HIV Tat also binds N-methyl-D-aspartate (NMDA) receptors, driving Ca^2+^ flux, hyper-activation and glutamate excitotoxicity in neurons [30,31,32,33]. In response to Tat, neurons also upregulate the generation of inhibitory synapses, which can alter the balance of excitatory versus inhibitory synapses [34], which can impact neuronal communication and information processing as observed in PWH. Interestingly, the impact of HIV Tat on neurons may be clade dependent as HIV Tat containing the Cys31Ser mutation found in PWH with HIV Clade C exhibits significantly less neurotoxic effects on NMDA receptors than those from Clade B without this mutation [35].

HIV envelope glycoprotein gp120 has been detected in both brain tissue and CSF of PWH [36] and exerts partly overlapping effects to that of HIV Tat through chemokine receptor signaling, sensitization of NMDA receptors and downstream pathways that impact dendritic retraction, synaptic loss, and susceptibility to apoptosis [37,38,39,40,41]. Specifically, HIV gp120 binds the chemokine receptors CCR5 and CXCR4 and can trigger neuronal signaling pathways including endocytic trafficking pathways associated with neurotoxicity [39,42]. Gp120 binding to chemokine receptors has been shown to induce endoplasmic reticulum stress and altered morphology, leading to Ca^2+^ depletion in neurons [43]. In vitro/in vivo studies demonstrated that HIV p120 can also induce mitochondrial damage and dysfunction in neurons, further contributing to neuronal damage [44]. In addition to effects within the brain cortex tissue, gp120 also contributes to HIV-associated pain and peripheral neuropathy via synapse degeneration and aberrant activation in neurons of the spinal cord dorsal horn.

The specific roles of other viral proteins including HIV nef, Vpu, Viral protein R (Vpr) and gp41 on neurons is comparatively ill-defined relative to that gp120 and Tat. Nef has been detected in the brain and CSF of PWH and SIV-infected non-human primates [45,46,47]. Ex vivo/in vitro studies have demonstrated that Nef can be released from infected cells in extracellular vesicles [48] and induce axonal and neurite damage [49]. Although the majority of evidence points to a bystander mechanism through activation of glial cells (discussed below). The HIV accessory protein Vpu has been shown to induce Caspase 3 activation and the accumulation of TAR DNA binding protein 43 in neurons during ectopic infection, which is characteristic of Alzheimer’s and amyotrophic lateral sclerosis [50]. Soluble Vpr has also been demonstrated to cause neuronal apoptosis [51].

### 3.2. Bystander Effects from Glial Activation and Systemic Inflammatory Mediators on Neurons

In addition to direct viral mediated neurotoxicity, bystander effects also contribute to neuronal dysfunction in PWH. While HIV does not directly infect neurons, HIV persists in glial cells including microglia and to a lesser extent astrocytes and pericytes in the brain [52,53,54,55]. Both viral infection and brain cell activation persist in the presence of ART which is detrimental to neuronal health. Activated microglia and infiltrating monocytes release cytokines, chemokines, reactive oxygen species, and lipid mediators that impair synaptic maintenance and alter neuronal excitability, increasing their vulnerability to excitotoxic and metabolic injury [56,57,58,59]. Specifically, HIV gp120 has been demonstrated to drive IL-1β production from glia that may sensitize NMDA receptor signaling and neurotoxicity [60,61]. Other viral proteins including HIV Tat can also induce a proinflammatory M1-like microglial phenotypes secreting IL-1β and TNF-α that may lead to neuronal injury and cell death [62,63]. This ongoing inflammatory response impacts neuron function even in the absence of neuronal cell death. The chronic low-grade immune activation shown to be present in the CNS despite effective ART treatment, has been shown to correlate with a reduction in synapse plasticity, and interferes with neurotransmitter homeostasis. This change in neuronal circuits resulted in slowed processing speed, impaired executive function, memory deficits, and altered motor control [64,65].

Astrocytes are important regulators of brain homeostasis, and although astrocytes have not been demonstrated to be productively infected by HIV, their dysfunction is recognized as a major contributor to HIV-related neuronal injury [66,67]. HIV-associated inflammation can impair the ability of astrocytes to take up glutamate [68,69,70,71], provide metabolic support [72], reduce antioxidants, and regulate the levels of extracellular ions; all of which increase neuronal susceptibility to excitotoxicity and energy failure [67,72,73,74,75,76]. Astrocytes also help preserve BBB integrity [5] and coordinate neurovascular coupling [77], and astrocytic dysfunction provides a mechanistic bridge between local neuroinflammation and broader tissue-level disturbances affecting neuronal health [78,79,80,81]. HIV Tat has been demonstrated to induce proinflammatory cytokine production from astrocytes with transient exposure shown to support hours of sustained cytokine release that can impact the surrounding cellular microenvironment, particularly neurons [82]. In astrocytes and microglia, Tat amplifies inflammatory responses and disrupts glutamate regulation [83,84].

Aberrant synaptic pruning has also been implicated in neuronal injury in PWH. Synaptic pruning is a normal function of microglia and astrocytes in order to remove unneeded connections between neurons. However, microglia and astrocytes have been demonstrated to enhance synaptic pruning of neuronal synapses in PWH. A recent study using HIV-gp120 transgenic mice found that animals displayed higher levels of microglial and astrocyte synaptic pruning relative to wild-type controls [85], supporting a mechanism by which viral protein directly contributed to damage of important pre-synaptic, and in some cases, post-synaptic terminals of neurons. Studies in mouse models have also demonstrated that Tat can drive microgliosis and synaptic pruning [86], further highlighting the contribution of viral protein mediated pathways in driving synaptic pruning. Interestingly, reduced synaptic density is observed clinically via specialized positron emission tomographic (PET) scanning even in ART-suppressed PWH [87], which raises the question as to whether these observations may relate to residual viral proteins in the brain, or whether alternate pathways drive synaptic pruning in ART-suppressed individuals.

### 3.3. Blood–Brain Barrier and Neurovascular Injury

BBB disruption is another important determinant of neuronal injury in HIV [79,88]. Compromised BBB integrity facilitates abnormal immune trafficking, permits exposure of neural tissue to circulating inflammatory mediators [81,89], and may impair the tightly regulated microenvironment required for normal synaptic transmission and metabolic support [79,88,90,91,92]. In older people with HIV, neurovascular injury likely interacts with HIV-specific mechanisms to amplify white matter damage, impair neuronal resilience, and complicate attribution of causality to HIV alone [92,93,94]. HIV gp120 can also activate endothelial cells of the BBB, driving dysfunction of the BBB and cytokine release which potentially enhances lymphocyte trafficking into the brain [95].

### 3.4. Metabolic and Mitochondrial Stress

Neuronal function in PWH is also impacted by mitochondrial dysfunction and altered brain metabolism [44,96]. Mitochondria are especially sensitive to inflammation and excitotoxic stress. When impaired, mitochondria produce less ATP, generate more reactive oxygen species, and become more likely to contribute to structural damage [44,67,96,97,98,99]. Magnetic resonance spectroscopy (MRS) studies showing lower levels of N-acetylaspartate and other metabolic changes suggest that neurons in HIV may be under metabolic stress even when there is no obvious tissue damage [100,101,102,103,104]. HIV Tat has been demonstrated to induce NLRP3 inflammasome activation in microglia via mitochondrial dysfunction and mitochondrial DNA leakage into the cytoplasm, resulting in the production of IL-1β and neuronal damage [105].

## 4. Structural and Functional Consequences for Neurons

### 4.1. Synaptic and Dendritic Injury

Synaptodendritic injury rather than massive neuronal loss is generally the earliest sign of impaired neuronal function in HIV infection [106,107,108]. Dendritic simplification, altered synaptic protein expression, and disruption of circuit connectivity can substantially impair cognition without marked neuronal cell loss [106,109,110,111]. This is particularly relevant in the context of ART-treated PWH, where subtle chronic synaptic pathology caused by ongoing HIV associated neuroinflammation likely explains persistent neurocognitive symptoms despite viral suppression [108,112,113].

Synaptic and dendritic injury may be the primary link between molecular damage and clinical symptoms. Viral proteins, inflammation, oxidative stress, and disrupted glutamate can all damage synapses and dendrites, which are energy-demanding and highly adaptable parts of neurons [109,111,114,115]. Damage at this level is sufficient to degrade neural network efficiency long before neurons are lost outright [106,116,117].

### 4.2. Axonal Injury and White Matter Disruption

Axonal integrity is also affected in PWH, as reflected by both imaging and fluid biomarker studies [118,119]. Analysis of neurofilament light chain (NfL) levels, surrogate measure of neuroaxonal injury, suggests that neuronal injury can occur across all stages of infection, from untreated HIV and HIV-associated dementia to ART-treated individuals with milder cognitive disorders [118,120,121,122]. White matter microstructural abnormalities analyzed on diffusion imaging demonstrate that HIV-related injury is not confined to cortical neurons but involves long-range connectivity and axonal maintenance [119,123,124,125]. This aspect of axonal injury is critical as brain function depends on both local synapses and long-range connections. Even minor axonal injury can contribute to slowed information processing and impaired executive function, which remain among the most common cognitive disorders in ART-treated PWH [126,127,128].

## 5. Confounding Contributors to Neuronal Injury

### 5.1. ART Regimens

ART has transformed the neurological consequences of HIV infection, but it has not fully normalized neuronal health. Effective ART reduces severe encephalitic pathology, lowers many inflammatory and injury biomarkers, and likely prevents a substantial burden of irreversible neuronal damage when initiated early [129,130,131]. However, ART does not eliminate all CNS inflammatory activity, does not fully reverse legacy damage, and may not completely suppress compartmentalized or transcriptionally active HIV in the brain and CSF [15,132]. This creates a therapeutic landscape defined by partial protection rather than complete restoration. Early ART appears especially important because it may limit the duration of uncontrolled CNS injury and reduce the risk of persistent structural and metabolic abnormalities later in life [4,133,134], although a study from the RV254 study found that early ART initiation during acute infection did not normalize levels of CSF NfL relative to those in seronegative controls [135]. At the same time, the persistence of milder impairment in well-treated cohorts argues that suppressing plasma viremia is necessary but insufficient for full preservation of neuronal function.

### 5.2. Aging, Comorbidity, and Heterogeneity

The primary issue for current neuroHIV research is that HIV-induced neuronal damage occurs in an older population with non-infectious comorbidities [136,137,138]. Cardiovascular disease, metabolic disorders, depression, sleep disorders, substance use, and previous brain injury can exacerbate neurological damage and confound the exact role played by HIV infection [139,140]. As a result, PWH represent a highly heterogeneous population where neuronal health is likely influenced by both viral mediated factors as well as host age, history of medication use, cardiovascular risks, and psychosocial factors [141,142].

Potential impacts of these comorbidities may account for the variation seen in cognitive phenotypes and biomarker results found in different studies [141,143,144,145]. Specifically, ART timing, CD4 nadir, history of encephalitic injuries, sex, geographical area, virus subtype, co-infection status, and assessment method would likely all influence an analysis of neuronal health and cognitive function [146].

### 5.3. Role of Co-Infections

Co-infection is a potential contributor of neuropathology in PWH. PWH who are co-infected with Hepatitis C (HCV) with significant liver disease have higher rates of cognitive impairment than those with HIV alone [147]. However, conflicting reports exist regarding whether HCV sero-status alone in the absence of liver disease elevates risk [148,149] with a large study from the CHARTER consortium not finding elevated incidence of neurocognitive impairment relative to HIV infection alone, although structure abnormalities in white matter were identified [150]. Cytomegalovirus (CMV) is ubiquitous in PWH and CMV coinfection is a known risk factor for cerebrovascular disease in PWH [151]. Specifically, within the brain, co-infection of CNS cells has been reported in PWH with AIDS and can contribute to encephalitis [152,153]. It is possible that CMV may have a tissue specific effect within the brain as CMV replication in the gut of PWH was demonstrated to drive epithelial activation and inflammation [154]. However, the full impact of HCMV reactivation is difficult to quantify systemically as while a study of 80 PWH (38 ART-treated and 42 ART-naïve) found that levels of plasma CMV IgG was associated with worse neurological outcomes in adjusted analyses [155], a recent report of a cohort of longitudinally followed PWH found that levels of CMV IgG in plasma did not correlate with measures of cognitive impairment [156]. Therefore, further studies are required to clarify the contribution of reactivation of CMV, and other pathogens for that matter, on cell activation in the brain and potential cognitive impairment in PWH.

Together, these comparisons are important for understanding HIV pathogenesis in the CNS as they show that neuronal health can be impacted either by direct infection or by injury to important support cells and the induction of an inflammatory environment.

## 6. Evidence and Tools for Assessing Neuronal Injury

To measure HIV-related neuronal injury, structural, biochemical, and clinical measures are used through two main approaches. Post-mortem tissue provides direct evidence of neuronal and synaptic damage, while neuroimaging and fluid biomarkers offer indirect but trackable evidence of injury in living individuals.

### 6.1. Neuropathological Evidence

Postmortem studies provide an opportunity to assess the cellular environment in the brain of PWH. Multiple studies of autopsy tissue have consistently linked neurocognitive impairment and HIV-related brain pathology with inflammatory markers such as IL-1β, TNF-α, IFNα, CD16+ monocytes and microglial and astrocyte activation, supporting a model in which neuronal injury occurs in regions of persistent innate immune activation [157,158]. Post-mortem tissue analysis has also made it possible to quantify neuronal loss, synaptic depletion, dendritic simplification, white matter injury, gliosis, and regional vulnerability, which are neurological patterns that cannot be established with the same level of confidence through live imaging techniques [54,159,160].

### 6.2. Neuroimaging in Living People with HIV

In living cohorts of PWH, magnetic resonance imaging (MRI)-based approaches provide non-invasive assessment of the structural and functional consequences of HIV-related injury. Structural and resting-state functional MRI studies in ART-suppressed PWH have shown lower gray matter volume and white matter abnormalities, that are suggestive of neuronal injury and tissue loss in these regions. These findings further suggest that detectable brain structural change persists despite ART-suppression of HIV [161,162,163]. The use of diffusion imaging adds sensitivity and allows detection of microstructural disruption affecting axons and white matter tracts, while functional imaging helps define how brain changes can result in altered network organization and cognitive decline [164].

MRS enables the detection of brain damage by monitoring metabolic correlates of neuronal damage, specifically identifying N-acetylaspartate (NAA) abnormalities that indicate HIV-related neuronal damage before it is visible on conventional scans [100,102,103,165]. Similarly, MRI, diffusion imaging, and MRS studies continue to detect structural and metabolic abnormalities in virally suppressed HIV cohorts, implying that neuronal function can remain incompletely restored despite long-term ART [102,132,166,167,168]. Together, these imaging methods support the idea that HIV-related neuronal injury in ART-treated PWH may be subtle, distributed, and chronic rather than grossly destructive.

### 6.3. CSF and Blood Biomarkers

The assessment of fluid biomarkers is particularly valuable because they allow repeated monitoring of neuronal health over time and can be incorporated into interventional studies. NfL in CSF and blood is an indicator of neuroaxonal injury and rises in severe disease, but it can also be abnormal in less advanced states and during primary infection [131]. NfL levels are associated with incidence of cognitive issues in PWH [169]. As NfL levels drop after starting ART and then stabilize, NfL is considered a dynamic marker of injury rather than just a reflection of early damage alone [170,171,172]. The utility of CSF biomarkers is also particularly informative when trying to understand mechanisms involving CNS compartment-related processes. Besides NfL, CSF biomarkers of astrocyte activation and intrathecal immune activation provide information regarding the presence or absence of a neuroinflammatory environment or dysfunction of the BBB in which the neuronal damage may occur. Whilst these are not direct surrogate measures relating to neuronal health and function, they may provide as assessment of the neuroinflammatory environment. For instance, there is a significant association between CSF YKL-40, CSF NfL, and indicators like albumin ratio, neopterin, and CSF HIV RNA levels [173,174]. Thus, these associations confirm the idea that poor neuronal state in HIV infection relates to glial activation and altered CNS immune status [170,171,173]. In ART-treated PWH it has been demonstrated that monocyte activation markers such as CSF neopterin, sCD163, and sCD14, remain associated with neurocognitive impairment despite viral suppression [170,175,176]; however, these data should be interpreted cautiously because biomarker abnormalities are not specific to HIV. Whether the detection of viral products in the plasma and/or CSF is prognostic of underlying neuropathology remains ill-defined. A study of ART-treated PWH detected HIV Tat in the CSF (36.8%) and CSF exosomes (34.4%) of individuals with levels associated with lower psychomotor speed and information processing [177]. However, CSF Tat levels were also associated with previous abuse of cocaine or amphetamines.

Taken together with post-mortem and mechanistic studies, these findings strengthen the view that treated HIV infection can leave the brain in a condition of persistent vulnerability characterized by incomplete recovery, ongoing low-grade injury, or both [15,129,132].

### 6.4. Interpretation and Limitations

There is also a need for proper interpretation of detection techniques as many of the in vivo markers are highly sensitive to HIV infection, yet non-specific to it. Factors such as aging, blood vessel diseases, kidney disorders, other diseases, drug use, sleep disturbance, depression, and previous immunosuppression can affect the neuroimaging and biomarker findings; thus, making it difficult to differentiate between HIV-induced neuropathy and other diseases. Therefore, the most important findings come from a multipronged approach including a combination of cognitive testing, neuroimaging, peripheral and CSF biomarkers, as well as postmortem findings where possible.

## 7. Implications for HIV-Cure Research

The impact of HIV on neuronal health is highly relevant to approaches for an HIV cure because the CNS has been shown to be a persistent viral reservoir in which ongoing viral transcription, immune activation, and altered glial states persist despite systemic HIV suppression [52,178,179]. However, the CNS is often not assessed in cure studies and presents challenges in delivery as many interventions have limited penetrance across the BBB. Consequently, little evidence of effective clearance/silencing of HIV infected cells in the CNS of PWH exists. Newer approaches including immunotherapeutics and have shown some promise at targeting the CNS reservoir in animal models; however, efficacy and safety in PWH is unclear [180,181]. If CNS reservoirs continue to drive inflammatory or transcriptional programs that may harm neurons, then cure strategies that ignore the brain risk underestimating residual ongoing damage. For instance, whilst Tat and other interventions have proved effective as latency-reversing agent in resting CD4+ T cell reservoirs [182,183], significant neurotoxicity concerns for the brain have been identified. Conversely, interventions that reduce systemic reservoirs without impacting neuronal or neuroimmune parameters may fail to address the CNS impact of persistent HIV disease.

This means that neuronal health and the implications of neuronal damage should be treated as both an outcome and a mechanism in the context of HIV-cure-focused research. The observed interplay of synaptic integrity, neuroaxonal injury, glial activation, BBB function, and cognitive performance provide a means by which one can assess whether the proposed cure interventions lower the damaging CNS activity or increase neuroinflammation. This is particularly important for latency-reversing, immune-modulating, and/or cell-targeted strategies that could have distinct effects in the CNS compared with peripheral blood.

## 8. Priorities for the Field

Several priorities emerge from the current literature. Firstly, longitudinal multiparameter studies are required that follow viral persistence, inflammatory signaling, neuronal biomarkers, and cognition over time. Second, we need a clearer understanding of measures that separate active neuronal injury from historical damage. Third, an improved ability to determine which aspects of neuronal dysfunction are temporary and reversible relative to permanent damage will aid in addressing appropriate therapeutic approaches. Moving beyond a primary focus on diagnoses of cognitive disorders toward a broader brain health framework may provide a more refined view of subclinical neuronal vulnerability, resilience, and improved considerations of aging and non-viral comorbidities. Such an approach appears more consistent with current evidence and may better align with the protecting brain function across the lifespan in PWH.

## 9. Conclusions

HIV exerts a durable and multifaceted impact on neuronal health in the brain, primarily through indirect mechanisms that target synapses, dendrites, axons, metabolism, and neural networks rather than through widespread productive neuronal infection. ART has dramatically reduced severe neurological disease, but it has not eliminated the legacy and ongoing processes that compromise neuronal integrity in many PWH. The most informative contemporary model is therefore one of chronic, heterogeneous, and only partly reversible neuronal stress that may be shaped by residual viral transcription, neuroinflammation, neurovascular dysfunction, and comorbidity.

This interpretation has practical consequences for both clinical and translational research. Preserving neuronal health should be treated as a core therapeutic outcome in treatment of HIV infection, not merely a secondary correlate of cognition. Moreover, CNS-informed endpoints should be integrated more systematically into studies of ART optimization, adjunctive neuroprotection, and HIV-cure strategies.

## Data Availability

No new data were created or analyzed in this study. Data sharing is not applicable to this article.

## Abbreviations

The following abbreviations are used in this manuscript:

ANI	Asymptomatic Neurocognitive Impairment
ART	Antiretroviral therapy
BBB	Blood–brain barrier
CMV	Cytomegalovirus
CNS	Central nervous system
CSF	Cerebrospinal fluid
CXCR4	C-X-C chemokine receptor type 4
gp120	HIV envelope glycoprotein 120
HABI	HIV-associated brain injury
HAND	HIV-associated neurocognitive disorders
HCV	Hepatitis C virus
HIV	Human immunodeficiency virus
IFNα	Interferon alpha
IL-1β	Interleukin-1 beta
MRI	Magnetic resonance imaging
MRS	Magnetic resonance spectroscopy
NAA	N-acetylaspartate
Nef	Negative regulatory factor
NfL	Neurofilament light chain
NMDA	N-methyl-D-aspartate
PET	Positron emission tomography
PWH	People with HIV
ROS	Reactive oxygen species
sCD14	Soluble cluster of differentiation 14
sCD163	Soluble cluster of differentiation 163
SIV	Simian immunodeficiency virus
Tat	Trans-activator of transcription
TNFα	Tumor necrosis factor alpha
Vpr	Viral protein R
Vpu	Viral protein U
